# Personal Growth and Motto Goals: Strengthening Emotion Regulation Ability via Affirmatory Metaphors Coaching

**DOI:** 10.5964/ejop.12031

**Published:** 2024-02-29

**Authors:** Elena Fischer, Christina Mühlberger, Julia Weber, Eva Jonas, Julius Kuhl, Markus Quirin

**Affiliations:** 1 Department of Psychology, Paris Lodron University of Salzburg, Salzburg, Austria; 2 Department of Psychology, Private University of Applied Sciences Goettingen, Goettingen, Germany; 3 ISMZ Zürich, Zurich, Switzerland; 4 Institute of Psychology, University of Osnabrueck, Osnabrueck, Germany; 5 Sport Psychology Unit, Technical University of Munich, Munich, Germany; London School of Economics, London, United Kingdom

**Keywords:** personality growth, coaching intervention, emotion regulation ability, neuroticism, extraversion

## Abstract

Interventions can foster personal growth. However, our understanding of the specific mechanisms for change and the types of interventions driving this growth process remains limited. In this study, we focused on emotion regulation ability as a potential mechanism. We examined the effects of an affirmation coaching intervention on changes in emotion regulation ability, an important facet of personality. In this coaching intervention, participants created a personal mantra/goal derived from a selected image and positive associations linked to this image (motto goals). This is considered to enhance emotion regulation abilities by internalizing self-stabilizing value. We assigned sixty-six participants to either this affirmation coaching intervention or one of two control coaching interventions: specific-goal versus indulgence coaching. Before and after each intervention, participants completed questionnaires. Only the affirmation coaching intervention significantly increased in adaptive aspects of personality. Notably, the affirmation coaching intervention increased emotion regulation ability, and this effect persisted even when controlling for extraversion and neuroticism. Furthermore, exploratory analysis showed that extraversion increased following the affirmation coaching, while neuroticism remained unchanged. Our results suggest that emotion regulation ability might be the key factor in personality growth. It could be more malleable and/or respond more strongly to short-term coaching, compared to neuroticism. Thus, the malleability of personality traits may not be an all-or-nothing phenomenon; rather, it could depend on the facet of emotion regulation ability. We discuss potential mechanisms of personality growth, distinguishing between emotion regulation and emotion sensitivity.

Contrary to a decades-long opinion on the relative stability of personality traits within personality psychology (Strelau, 1983), a broad meta-analysis demonstrated that personality, especially neuroticism and extraversion, can be changed through interventions (Roberts et al., 2017). Neuroticism is a major personality trait that reflects the experience of negative states relating to anxiety, depression, or vulnerability (McCrae & Costa, 2013). Individuals with high levels of neuroticism inhibit arousal more slowly and are aroused more quickly following aversive stimuli (Rusting & Larsen, 1997). Extraversion, in contrast, has been associated with a sensitivity to positive experiences and happiness (Rusting & Larsen, 1997), and individuals high in extraversion may thus regulate negative emotions by approaching positive experiences (Ng & Diener, 2009).

## Personality Growth Interventions

Therapeutic and non-therapeutic interventions show personality changes with similar effect size (Roberts et al., 2017). This is interesting because any change demonstrated by non-clinical interventions is a stronger indication of real improvement beyond a return to baseline of a healthy psyche. Within interventions, discrepancy awareness, resource activation, awareness of beliefs/motives, and practicing new behaviours are relevant factors for personality growth^1^1We refer to ‘personality growth’ as adaptive changes in personality traits. ‘These adaptations might encompass the integration of negative experiences as well as improvements in needs and goal attainment, which are typically considered as a basis for enhancements in happiness’ (Quirin et al., 2023). (Stieger et al., 2021). Also, specific goals and plans appear to be important for personality growth (Hudson & Fraley, 2015).

However, it is unclear which underlying mechanisms drive this effect and which types of interventions are especially effective—questions that have not sufficiently been answered by previous studies (Roberts et al., 2017). Still, such research is highly interesting because of the high prevalence of mental illness and the alignment and optimization of treatments. As we will argue, the ability to regulate negative emotions may constitute such a mechanism, and that an intervention that uses affirmations may have the potential to increase an individual’s emotion regulation ability (ERA). Thus, theoretical implications from the current study encompass an enhanced comprehension of the mechanisms underlying personal growth, while practical implications pertain to recommendations for coaches.

Self-affirmation is a basic motivator that promotes self-worth and psychological well-being (Pandey et al., 2023). It involves striving for self-integrity by being reminded of one's own self-esteem and other self-resources (Pandey et al., 2021). Consequently, self-affirmation entails the act of reflecting on cherished values or one's own positive attributes, resulting in adaptive cognitive and behavioral outcomes. The applied affirmation coaching (see motto goals, Storch et al., 2011) addresses relevant components of interventions for personality growth: Personal resources and positive emotions are focused on in addition to conscious motives. Since a large part of emotion regulation processes operate unconsciously (Koole & Rothermund, 2011), unconscious needs are also addressed in this coaching. Moreover, affirmations have already been found to influence emotion-regulation ability (Storch et al., 2011) and the self-system is involved in the formation of affirmations by activating self and emotion regulation (Quirin, Bode, & Kuhl, 2011). The term ‘self-system’ pertains to an individual's own convictions derived e.g., from personal values, as opposed to being biased by external forces such as other people. We anticipate that the affirmation coaching will yield personality growth due to an alignment of the mentioned components with change factors for personality growth interventions (Allemand & Flückiger, 2017).

Here we follow a functional, process-oriented approach according to which traits are considered relatively stable tendencies to adopt cognitive or affective processes (Quirin et al., 2020). Such an approach is oriented towards distinguishing between process-related tendencies such as emotion sensitivity versus emotion regulation (Quirin et al., 2023).

Previous research used extraversion and neuroticism as proxies for emotional sensitivity (Rusting & Larsen, 1997). In contrast to the process-oriented approach, factor-analytical models (e.g., the five-factor model) do not include personality components (domains or facets) explicitly expressing ERA, which does not contradict the possibility that ERA is a distinct trait as items submitted to factor analysis may not have been designed to express regulation (Quirin et al., 2020). In fact, previous studies demonstrated that the interaction between high emotional sensitivity and high ERA, which are typically negatively correlated, predicts well-being, over and above low levels of emotional sensitivity (Baumann et al., 2007). Accordingly, emotional sensitivity (as for example approximated by extraversion or neuroticism scales) and ERA are distinct traits.

## Emotion Regulation Ability

ERA, the self-regulation of emotions, is considered the potential to autonomously modify the affective response to salient (unpleasant) stimuli and is a central and distinct personality trait (Koole, 2009).

ERA has been related to extraversion and neuroticism. Extraversion has strongly been associated with a sensitivity to positive affective states and neuroticism more readily trigger negative affective states (Rusting & Larsen, 1997). Neuroticism was related to reduced usage of reappraisal across different study designs (Hughes et al., 2020). Moreover, high neuroticism was associated with low adaptability and reduced persistence and with more emotion regulation strategies that are typically considered maladaptive (Barańczuk, 2019). Extroverts are more likely to use emotion regulation strategies that involve situation modification or cognitive reappraisal (Barańczuk, 2019). Ng and Diener (2009) found that dysfunctional ERA, i.e., suppression and repression, mediate the link between neuroticism and negative emotions. Functional ERA, i.e., reappraisal and acceptance, mediate the association between extraversion and positive emotions.

Additionally, self-regulatory and self-focused personality constructs, such as self-esteem, as well as factors like social desirability, job attitudes, and performance, are positively correlated with ERA (Diefendorff et al., 2000). There are also findings on hormones that show that ERA predicts the oxytocin-cortisol interaction (Quirin, Kuhl, & Düsing, 2011) and the interaction of left-frontal activation shift (EEG) with cortisol response to a social-evaluative threat (Düsing et al., 2016). Furthermore, ERA was found to be related to the functional network of the amygdala (Schlüter, Fraenz, Pinnow, Friedrich, et al., 2018) and to increased left hemispheric prefrontal asymmetry under the control of behavioral activation or inhibition (Haehl et al., 2021).

Furthermore, the following differentiation can be made. ERA refers to individual differences in regulating emotions once emotions are aroused (Koole, 2009). By contrast, neuroticism and extraversion may refer to individual differences in both emotion generation (emotional sensitivity) and emotion regulation (emotion regulation emerge after emotion generation). However, ERA has not yet been studied as the malleable component in personality growth.

Evidence show that ERA may be more malleable than emotional sensitivity. For example, neuroticism is moderately inheritable, whereas ERA appears to be more socialized (McRae et al., 2017; Suri et al., 2018). In general, the body of studies also indicates that ERA is learnable (Morris et al., 2007). Because of this relative malleability, it is likely to assume that it is ERA that ultimately drives personality growth. Consequently, potential intervention effects may specifically affect ERA changes.

## Present Research and Hypotheses

We investigate ERA as a potential underlying variable influencing personality growth and applied an affirmation coaching (see motto goals, Storch et al., 2011). A third of the participants were assigned to this affirmation coaching, and the other participants received one of the two control interventions (one-third received a specific-goal coaching and the remaining one-third received an indulgence coaching).

Our hypothesis are as follows: First, we predicted that an affirmation coaching significantly increases participants' ERA compared with the control interventions (Hypothesis 1). Next, and most importantly, we predicted that this ERA improvement results from changes in the regulation of emotions once they are aroused rather than emotion generation or sensitivity (Hypothesis 2). We therefore expect that if we account for extraversion and/or neuroticism as covariates, the improvement in ERA remains significant.

## Method

We used a 3 (affirmation coaching (experimental), specific-goal coaching (control), indulgence coaching (control)) × 2 (pre-, and post-conditions) research design. Consequently, participants in the experimental and control conditions were tested for ERA, neuroticism, and extraversion before and after receiving coaching.

### Participants

We conducted a power analysis using G*Power 3.1. (Faul et al., 2007). The required sample size is 66 persons (assuming medium effect size *f* = 0.25, 1-β = 0.80, and α = 0.05). A total of 66 participants (50 female, 42 students; *M*_age_ = 32.9 years, *SD* = 13.7) from Osnabrueck, Germany and the surrounding area participated in this study. They were recruited via notices at the University of Osnabrueck and via advertisements in a local newspaper.

Participants were randomly assigned to six groups (two coaching groups for each condition). Of the 70 participants initial registered, four did not show up for the coaching and four changed coaching groups due to time constraints before the coaching started. The affirmation coaching had 24 participants (17 female; *M*_age_ = 33.7 years, *SD* = 14.0), the specific-goal coaching 23 (18 female; *M*_age_ = 32.4 years, *SD* = 13.6), and the indulgence coaching 19 (15 female; *M*_age_ = 32.4 years, *SD* = 14.1). The Ethics Committee of the University of Osnabrueck approved the study.

### Inclusion and Exclusion Criteria

Inclusion criteria was a motivation to participate in coaching that reflected a genuine interest in personal change. A glucose measurement, which, though not pertinent to the current paper, resulted in the following exclusion criteria: diabetes, pregnancy, and the use of permanent medication.

### Procedure and Coachings

**Figure 1:**
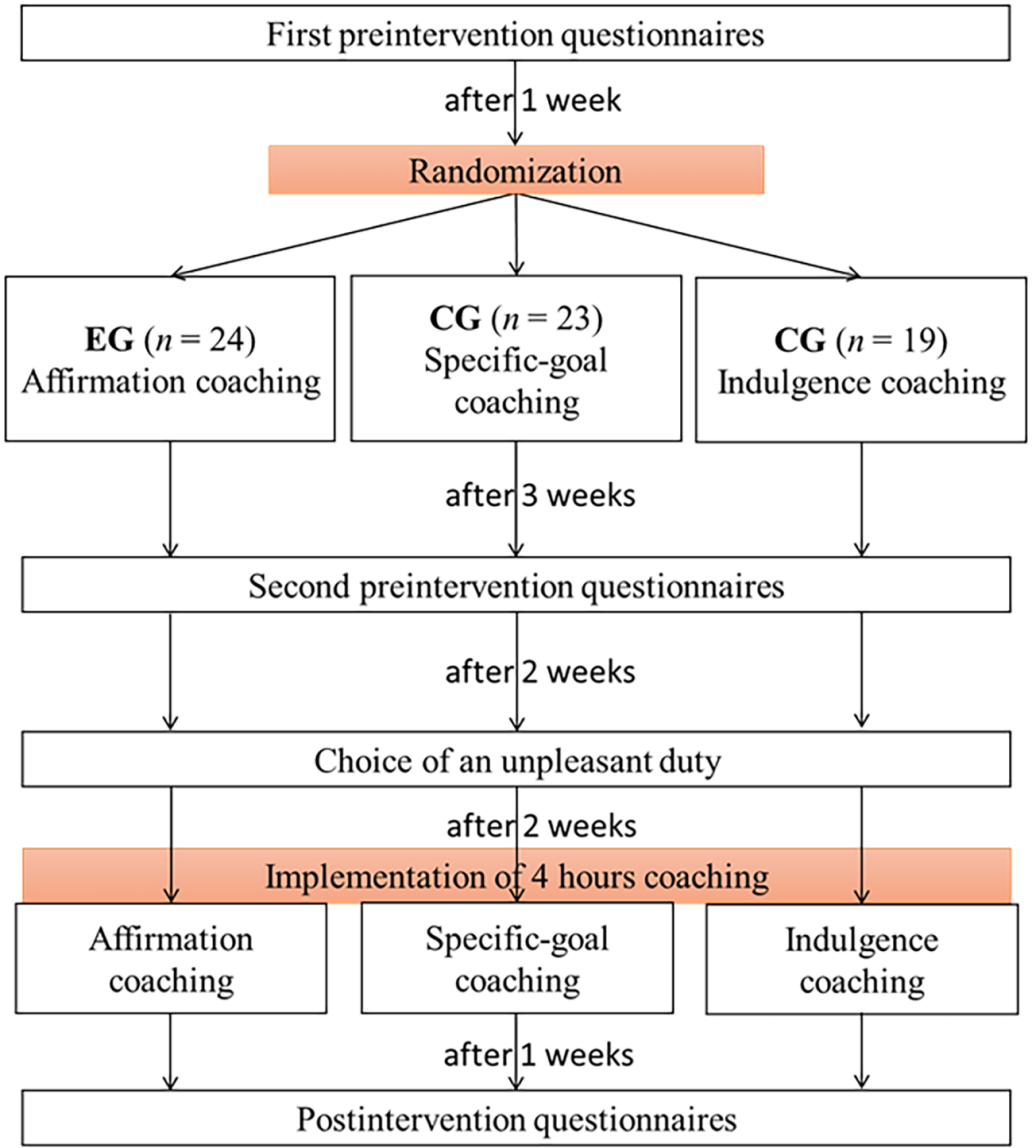
Procedure of the Study *Note.* EG = experimental group; CG = control group.

A battery of questionnaires including those on ERA, neuroticism and extraversion, were completed twice before the coaching to determine a baseline score of the participants (see Figure 1). Before the start of the intervention, participants reported a personal unpleasant duty to the study administration, i.e., a regular or even daily activity that is performed reluctantly. We worked on unpleasant duties for reasons of uniformity or standardization and because of the given discrepancy between the actual self and the ideal/ought self. Further, in performing an unpleasant duty, it is important to be able to regulate one’s emotions.

In the coachings, individuals learned how to formulate either an affirmation, a specific goal or they indulged in positive future-state fantasies related to their unpleasant duty. The three group coachings, i.e., groups of people who acquire or improve personal skills through exchange on the principle of self-help, were conducted in a 4-hour face-to-face coaching of 9 to 13 participants.

#### Affirmation Coaching

In the affirmation coaching (Storch et al., 2011), participants selected an image that serves as a resource for their unpleasant duty. Afterwards, positive associations to the pictures of each participant were formed in small groups. Working individually in a stepwise process, an affirmation was then formulated on these positive associations. For example, one participant selected a photo of an orange and created the affirmation ‘*By virtue of the spring sun I mature to a firm and sweet fruit*’ (unpleasant duty: ‘*Prepare and rework courses*’).

#### Specific-Goal Coaching

Formulating specific goals has been widely studied and proven to be effective regarding goal attainment (Locke & Latham, 1990). However, in comparison to affirmations, specific goals lead to low goal achievement and lower intrinsic motivation (Bruggmann, 2003).

In the specific-goal coaching, a high specific goal was formed (Locke & Latham, 1990). The participants initially divided their unpleasant duty into five subgoals. Then they rated these subgoals by difficulty (0 = not difficult, 100 = extremely difficult) and selected one subgoal from the range between 70 and 90. This goal was specified using questions (e.g., ‘*When will you implement your goal?*’). Subsequently, the participants summarized what they had identified into a clearly formulated goal. An example of a specific goal is ‘*Sunday and Thursday I will go jogging in the park for 1h right after work, even if the weather is bad, because I will feel better afterwards*’ (unpleasant duty: ‘*Exercise regularly*’).

#### Indulgence Coaching

Since the literature shows that goal setting is particularly relevant for personality growth (Hudson & Fraley, 2015), and goals are formulated in both affirmation coaching (affirmations) and specific-goal coaching (action goals), we implemented a further control intervention. In this indulgence coaching, positive affect and personal resources are triggered through positive future-state fantasies. This provides a precipitate emotional reward for achieving goals, which does not lead to direct behavioural activation (Oettingen, 2012).

In the indulgence coaching, future-related, spontaneous, and positive simulations were formed for the unpleasant duty, ignoring negative aspects of reality (Oettingen, 2012). In group discussions, positive future fantasies were exchanged. This was followed by a half-hour imagination of the future in which participants were guided into personal positive future fantasies. The future fantasies were then recounted in the plenary. Next, in a written reflection exercise, the participants wrote down their positive future fantasies. The participants then drew a picture of their most beautiful future fantasy with coloured crayons. An example of a positive future fantasy is ‘*After writing the thesis, I feel at peace with myself, which leads me to be calm in my studies and life, and which makes me successful*’ (unpleasant duty: ‘*write bachelor thesis*’).

The coachings were conducted by two coaches, in which each coach conducted each coaching once. The coaches were blinded to the theoretical concepts and the hypotheses of the study. The post data collection took place one week after the coachings.

### Measures

#### Neuroticism and Extraversion

The two subscales ‘extraversion’ and ‘neuroticism’ of the German version of the NEO-FFI (Borkenau & Ostendorf, 1993) were used for the present study. The scale measures the extent which statements apply to one in general using a 5-point Likert scale ranging from *Strongly disagree* (0) to *Strongly agree* (4). In the current study, the subscale ‘extraversion’ and ‘neuroticism’ had an internal consistency of .84 (12 items, e.g., ‘I enjoy having a lot of people around me’) and .89 (12 items, e.g., ‘I often feel inferior to others’), respectively. The test-retest reliability of the two scales, measured at the first survey and after the intervention, show for ‘extraversion’ an *r*_RT_ = .88, *p* < .001 and for ‘neuroticism’ an *r*_RT_ = .88, *p* < .001.

#### Emotion-Regulation Ability

The German HAKEMP measures action control in three scales (Kuhl, 1994; for English version see ACS). We used the 12-item subscale measuring action orientation after failure vs. preoccupation. This subscale measures the degree to which individuals disengage from negative experiences and concomitant emotions. Each item contains a description of a situation, followed by two alternative answers (e.g., ‘When I’ve worked for weeks on one project and then everything goes completely wrong …’; (a) ‘… it takes me a long time to get over it’ [state-oriented approach]; (b) ‘… it bothers me for a while but then I don’t think about it anymore’ [action-oriented approach]). In the present study, the internal consistency is α = .86. The test-retest reliability, measured at the first survey and after the intervention, shows an *r*_RT_ = .76, *p* < .001.

### Statistical Analysis

The statistical analysis was performed using RStudio (2022) Version 4.0.5 and IBM SPSS Statistics (2019) Version 25. The alpha error level was set at 5%. After excluding one outlier and checking for normal distribution (Shapiro-Wilk test), differences in the two baseline measurements and between the coachings in the baseline measurements were examined using ANOVA. Then, we calculated the mean of the two pre-measurements, which we used as the baseline value for all further calculations. To investigate associations between variables, Pearson's correlation coefficients were calculated. Using the package lme4 in RStudio, mixed-effects models were performed to calculate differences between the coachings and to explore associations between variables. For this purpose, we set crossed random effects for participants and coded the fixed effects of condition (affirmation, specific-goal, indulgence coaching) and the time of measurement (before, after coaching) as factors. Due to repeated measurements, we included random intercepts to account for the by-participant dependencies. To account for prior differences between participants, we controlled for the baseline scores (dV). We compared the models using the AIC and determined normal distribution and homoscedasticity using residual plots (sjPlot package). To fit the models, we used restricted maximum likelihood estimation. *p*-values were determined using likelihood ratio tests of the model with the interaction effect (Group × Time) or the covariate(s) against the model without this interaction effect or without the covariate(s) (see Appendix A and Appendix B). The corresponding effect sizes ($\eta_{p}^{2}$) were interpreted according to Cohen (1988), with $\eta_{p}^{2}$ = 0.01 as a small effect, $\eta_{p}^{2}$ = 0.06 as a medium effect, and $\eta_{p}^{2}$ = 0.14 as a large effect.

## Results

Table 1 shows the descriptive findings. Non-significant results were found in the Shapiro-Wilk test, thereby affirming the assumption of a normal distribution, except for the post-measurement of neuroticism in the specific-goal coaching.

**Table 1:** Means and Standard Deviations for Each Variable and Each Coaching Intervention

Variable	Affirmation Coaching			Specific-Goal Coaching			Indulgence Coaching		
	T1	T2	T3	T1	T2	T3	T1	T2	T3
ERA	4.75 (3.33)	5.33 (3.61)	6.42 (3.45)	6.05 (3.44)	6.18 (4.01)	6.09 (3.71)	5.11 (3.38)	4.74 (3.51)	4.74 (3.74)
Extraversion	28.54 (6.14)	28.25 (7.24)	29.67 (5.80)	28.68 (7.75)	28.55 (6.69)	28.73 (7.13)	27.53 (6.49)	27.37 (8.15)	26.84 (8.19)
Neuroticism	21.04 (8.01)	19.83 (8.44)	19.83 (8.75)	21.45 (9.63)	21.14 (9.73)	22.14 (8.52)	23.37 (8.06)	24.58 (8.59)	23.21 (8.82)

*Note*. T1 = pre-coaching (8 weeks before coaching); T2 = pre-coaching (4 weeks before coaching); T3 = post-coaching (1 week after coaching); ERA = emotion regulation ability.

We found no significant differences in the pre-measurements among the three coaching conditions. Residual errors and random effects showed normal distributions for all variables. ERA and extraversion correlate significantly positively (*r* = .29, *p* = .02), whereas ERA and neuroticism do not correlate significantly (*r* = -.18, *p* = .16).

Mixed models analyse showed Group × Time interactions for ERA. Specifically, ERA increased following affirmation coaching but remained unchanged after both specific-goal coaching and indulgence coaching (see Table 2). Consistent with our second hypothesis, these significant interactions persisted when we controlled for changes in extraversion (i.e., difference score T2-T1). Furthermore, we controlled for change in neuroticism (i.e., difference score T2-T1). The Group × Time interactions for ERA once again remained statistically significant. Furthermore, these interactions persisted even after controlling for both extraversion and neuroticism.

**Table 2:** Group×Time Interactions of the Mixed Models

Interaction	Est/Beta	*SE*	95% CI	*t*	*p*	$\eta_{p}^{2}$
ERA as dV						
Coaching 1 × Time	-1.40	0.53	[-2.41, -0.38]	-2.64	0.011	0.05
Coaching 2 × Time	-1.56	0.55	[-2.62, -0.50]	-2.84	0.005	0.06
ERA as dV + Extraversion as cV						
Coaching 1 × Time	-1.40	0.53	[-2.40, -0.39]	-2.66	0.009	0.10
Coaching 2 × Time	-1.56	0.55	[-2.60, -0.51]	-2.83	0.005	0.11
ERA as dV + Neuroticism as cV						
Coaching 1 × Time	-1.40	0.53	[-2.41, -0.39]	-2.65	0.009	0.05
Coaching 2 × Time	-1.56	0.55	[-2.61, -0.51]	-2.84	0.005	0.07
ERA as dV + Neuroticism and Extraversion as cV						
Coaching 1 × Time	-1.40	0.53	[-2.43, -0.37]	-2.66	0.009	0.05
Coaching 2 × Time	-1.56	0.55	[-2.63, -0.49]	-2.85	0.005	0.07
Extraversion as dV						
Coaching 1 × Time	-1.16	0.84	[-2.77, 0.46]	-1.37	0.172	0.03
Coaching 2 × Time	-1.88	0.88	[-3.56, -0.19]	-2.14	0.034	0.07
Extraversion as dV + ERA as cV						
Coaching 1 × Time	-1.16	0.84	[-2.80, 0.48]	-1.38	0.169	0.02
Coaching 2 × Time	-1.88	0.87	[-3.58, -0.17]	-2.15	0.033	0.04
Neuroticism as dV						
Coaching 1 × Time	1.45	1.02	[-0.55, 3.44]	1.41	0.160	0.02
Coaching 2 × Time	-0.16	1.06	[-2.23, 1.91]	-0.15	0.880	0.00

*Note*. Coaching 1 = affiliation vs. specific-goal coaching; Coaching 2 = affiliation vs. indulgence coaching; Time = Pre- vs. Postcoaching; ERA = emotion regulation ability; dV = dependent Variable; cV = control Variable; *df* = 62.

### Exploratory Analysis

We identified one significant Group × Time interaction for extraversion, indicating that extraversion increased following the affirmation coaching compared to the indulgence coaching. We then controlled for change in ERA (difference score T2-T1) and found that this interaction remained significant. Conversely, for neuroticism, we did not observe any significant interactions.

## Discussion

This study contributes to the discussion on the malleability of personality by examining the association between ERA and extraversion and neuroticism within this context. We predicted and found that an affirmation coaching significantly increases participants' ERA. Moreover, we hypothesized that after controlling for extraversion and/or neuroticism, the significant change in ERA persist. Our data also confirmed this hypothesis, indicating that the improvement in ERA is not attributable to changes in emotion generation but to changes in emotion regulation.

The present experiment is promising, because it highlights that it is plausible that ERA could be the key factor in personality growth. Considering this, our results suggest that there is no either-or in terms of the malleability of personality, but it depends on the component of ERA. This assumption could explain contradictory evidence on the changeability of personality (Roberts et al., 2017; Strelau, 1983). For instance, it is possible to change how well one copes with a particular stressful situation (ERA), but not how strongly one reacts to it.

In line with this, ERA overlaps with but also differs from extraversion. Both are associated with high positive mood (Larsen & Ketelaar, 1989), which may have had an impact on the increase in these (positive affect is a central feature of the affirmation coaching). For example, it has been found that ERA leads to a shift in attention away from negative stimuli in major depression (Düsing et al., 2021). Extraversion, in contrast, relates to sensitivity to incentives (Rusting & Larsen, 1997). Additionally, ERA correlates low to moderately positively with extraversion (Kuhl, 1984).

The effects we found align with existing findings. Evidence show that ERA may be more malleable than emotional sensitivity (McRae et al., 2017; Suri et al., 2018). Other studies demonstrate that interventions can increase ERA (Gross, 2015). Treatments that do not directly target ERA, as well as those specifically designed to increase ERA, have positive effects on ERA (Sloan et al., 2017).

Despite controlling for ERA, the significant increase in extraversion was still observed after the affirmation coaching. The group setting and the interaction with other people (cf. sociability, activity) may have had a particularly positive effect on the increase of extraversion. Positive affect could also be responsible: On the one hand, extraversion may increase because more positive affect is present, and on the other hand, increased positive affect may be due to an increase in extraversion. In general, the increase in extraversion suggests personality growth, as extroverted behaviour leads to a significantly higher well-being compared to introverted behaviour (Margolis & Lyubomirsky, 2020). It must be mentioned, however, that greater extraversion is not always better, especially when it comes to very high levels, which was not the case in the present study.

Several studies have reported the beneficial effects associated with the practice of self-affirmations. Consistent with our findings, prior research has shown the efficacy of self-affirmation interventions, specifically in increasing positive emotional states while mitigating negative ones (Pandey et al., 2021, 2023).

As expected, individuals who received indulgence or specific-goal coaching did not change in ERA, extraversion, and neuroticism. This can be ascribed to the intervention's failure to target crucial change factors that influence personality growth. Regarding Allemand and Flückiger's (2017) change factors, the affirmation coaching directly addresses the mechanisms of resource activation and motivation clarification, in contrast to the control conditions. It could therefore be assumed that these two factors apply to the mechanism of emotion regulation.

The results are particularly surprising considering the brevity of the intervention (four hours) as intervention intensity plays a role in how effective an intervention is (van Agteren et al., 2021). Interventions that lasted less than a month showed less personality growth than those that lasted longer (Roberts et al., 2017). As such, future studies using a longer affirmation intervention could find changes in neuroticism. This would be desirable because neuroticism is strongly positively correlated with depression and anxiety (Jylhä & Isometsä, 2006). Another explanation for no improvement in neuroticism could be that the affirmation coaching did not target negative emotions. Instead, it focused on personal values, resources, and positive emotions. If it had focused on changing negative emotions, the results on neuroticism might have been different. In addition, the sample might have been a contributing factor, as the mean score of neuroticism was already low before the intervention (*M* = 21.86, *SD* = 8.48; in a validation study by Körner et al., 2008, *M* = 19.47, *SD* = 1.62). Higher neuroticism scores before the intervention might have resulted in a significant reduction in neuroticism.

### Limitations and Future Direction

Research has shown that self-selection makes a difference in intervention studies (Lyubomirsky et al., 2011). Our study was advertised as a self-management coaching program, but not as an intervention for personality growth. In other words, participants in the present study where motivated to increase their ERA. Recruiting participants who wanted personality growth might have yielded different results.

We focused on the immediate effects. This limits the ability to infer actual personality change from this study. In terms of practice, it is certainly also of interest to examine the long-term stability of change. Studies have already shown that personality growth by therapy remain stable for a period of more than one year (Roberts et al., 2017). This would be particularly interesting regarding future studies using the affirmation coaching, as there is already evidence that affirmations show longer-term effects on ERA (Fröhlich et al., 2012).

Despite the advantages of a high ERA, it should not be forgotten that there are also advantages of low ERA. For example, low ERA prevents premature actions and is associated with an increased risk perception (Koole et al., 2005). Next, other mechanisms could also be responsible for the effects found. A warm appreciation of the client in the affirmation coaching could impact the outcomes.

Desirability for future research would be an idiographic approach to achieve valid conclusions at the individual level (as within-person personality does not need to match between-person personality, Renner et al., 2020). This approach opens an opportunity to better examine the effects of context and time. Findings could subsequently be used to target personality growth through personalized interventions. Furthermore, future studies should investigate further interindividual differences, such as gender differences (Schlüter, Fraenz, Pinnow, Voelkle, et al., 2018).

### Conclusion

The present study provides empirical evidence of changes in personality scale scores that might indicate personality growth. Affirmation coaching increased ERA, and this effect persisted even when controlling for extraversion and neuroticism. Additionally, extraversion increased after the affirmation coaching, while neuroticism remained unchanged. Our results point to ERA being an important mechanism in the context of personality and coaching. Examining the functional mechanisms underlying personality, as we have explored by examining sensitive and regulatory aspects, has the potential to reconnect personality psychology with neighbouring disciplines, such as clinical psychology.

## Appendix A

**Table A1:** Model Building Processes for the Linear Mixed Models

Sampling units		*N* total observations = 130 *N* subjects = 65		Random effect subjects	Model fit				LRT test against nested	
Model specification	Model name	Nested / simpler model	Fixed effects added							
					AIC	BIC	LL	*df*	*df*	X2
Emotion regulation										
Random Effects only	Null	—	—	intercepts	620	629	-307	3		
Fixed Effects two-way interactions + main effect	Group x Time of Measurement + ERA_Baseline	Null	Group x Time of Measurement + ERA_Baseline	intercepts	441	467	-212	9	6	191.00***
Fixed Effects two-way interactions + main effects	Group x Time of Measurement + ERA_Baseline + E	Fixed Effects two-way interactions	+ E	intercepts	400	469	-210	10	1	2.68
Fixed Effects two-way interactions + main effects	Group x Time of Measurement + ERA_Baseline + N	Fixed Effects two-way interactions	+ N	intercepts	441	470	-211	10	1	1.69
Fixed Effects two-way interactions + main effects	Group x Time of Measurement + ERA_Baseline + E + N	Fixed Effects two-way interactions	+ E + N	intercepts	441	473	-210	11	2	3.78
Extraversion										
Random Effects only	Null	—	—	intercepts	761	769	-377	3		
Fixed Effects two-way interactions + main effect	Group x Time of Measurement + E_Baseline	Null	Group x Time of Measurement + E_Baseline	intercepts	562	588	-272	9	6	210.00***
Fixed Effects two-way interactions + main effects	Group x Time of Measurement + E_Baseline + ERA	Fixed Effects two-way interactions	+ ERA	intercepts	562	590	-271	10	1	2.63
Neuroticism										
Random Effects only	Null	—	—	intercepts	813	821	-403	3		
Fixed Effects two-way inter-actions + main effect	Group x Time of Measurement + N_Baseline	Null	Group x Time of Measurement + N_Baseline	intercepts	618	639	-297	9	6	212***

*Note*. AIC = Aikake Information Criterion; BIC = Bayesian Information Criterion; LL = LogLikelihood; *df* = degrees of freedom; LRT = Likeilhood Ratio Test; X2 = Chi-square; ERA = Emotion Regulation Ability; E = Extraversion; N = Neuroticism; Model Specification = the current model and what it includes or what was added to the previous model; Nested / simpler model = the model against which the current one is being tested, using the Model Name as a label; Fixed effects added = the fixed effects which have been added.

****p* < .001.

## Appendix B

Final models of the linear mixed models^2^ 2 *p*-values for fixed effects have been calculated using Satterthwaites approximations. Confidence Intervals have been calculated using the Wald method. In all models ERA = Emotion Regulation Abilities, E = Extraversion, N = Neuroticism, Group 2 = affiliation vs. specific goal coaching, and Group 3 = affiliation vs. indulgence coaching.

**Table B1:** Emotion Regulation

Variable	Est/Beta	*SE*	95% CI	*t*	*p*	Variance	*SD*
Fixed Effects							
Intercept	0.22	0.31	[0.37, 0.81]	0.71	.480		
Group 2	0.05	0.38	[-0.67, 0.76]	0.12	.902		
Group 3	-0.01	0.39	[-0.75, 0.74]	-0.01	.989		
Time	1.38	0.37	[0.67, 2.08]	3.76	< .001		
Emotion regulation Baseline	0.96	0.03	[0.89, 1.02]	29.14	< .001		
Group 2 X Time	-1.40	0.53	[-2.41, -0.38]	-2.64	.009		
Group 3 X Time	-1.56	0.55	[-2.62, -0.50]	-2.84	.005		
Random Effects							
Participant (Intercept)						0.00	0.00
Residual						1.60	1.27

*Note.* Model equation: ERA ~ Group * Time + ERA_Baseline + (1 | VpnCodeScreening). Model fit: R^2^ marginal = 0.87, R^2^ conditional = 0.87.

**Table B2:** Emotion Regulation (Including Extraversion as Covariate)

Variable	Est/Beta	*SE*	95% CI	*t*	*p*	Variance	*SD*
Fixed Effects							
Intercept	0.12	0.31	[-0.48, 0.71]	0.37	.712		
Group 2	0.11	0.38	[-0.60, 0.83]	0.31	.760		
Group 3	0.11	0.39	[-0.64, 0.87]	0.29	.772		
Time	1.38	0.36	[0.66, 2.09]	3.79	< .001		
Emotion regulation Baseline	0.96	0.03	[0.68, 1.02]	29.36	< .001		
Extraversion	0.06	0.04	[-0.01, 0.14]	1.60	.113		
Group 2 X Time	-1.40	0.53	[-2.40, -0.39]	-2.66	.009		
Group 3 X Time	-1.56	0.55	[-2.60, -0.51]	-2.85	.005		
Random Effects							
Participant (Intercept)						0.00	0.00
Residual						1.58	1.26

*Note.* Model equation: ERA ~ Group * Time + ERA_Baseline + E + (1 | VpnCodeScreening). Model fit: R^2^ marginal = 0.87, R^2^ conditional = 0.87.

**Table B3:** Emotion Regulation (Including Neuroticism as Covariate)

Variable	Est/Beta	*SE*	95% CI	*t*	*p*	Variance	*SD*
Fixed Effects							
Intercept	0.19	0.31	[3.40, 0.78]	0.62	.538		
Group 2	1.11	0.38	[-0.62, 0.83]	0.28	.781		
Group 3	-0.01	0.39	[-0.75, 0.73]	-0.03	.976		
Time	1.38	0.36	[0.68, 2.07]	3.77	< .001		
Emotion regulation Baseline	0.96	0.33	[0.89, 1.02]	29.23	< .001		
Neuroticism	-0.04	0.33	[-0.10, 0.02]	-1.26	.208		
Group 2 X Time	-1.40	0.53	[-2.41, -0.39]	-2.65	.009		
Group 3 X Time	-1.56	0.55	[-2.61, -0.51]	-2.84	.005		
Random Effects							
Participant (Intercept)						0.00	0.00
Residual						1.60	1.26

*Note.* Model equation: ERA ~ Group * Time + ERA_Baseline + N + (1 | VpnCodeScreening). Model fit: R^2^ marginal = 0.87, R^2^ conditional = 0.87.

**Table B4:** Emotion Regulation (Including Extraversion and Neuroticism as Covariate)

Variable	Est/Beta	*SE*	95% CI	*t*	*p*	Variance	*SD*
Fixed Effects							
Intercept	0.10	0.31	[-0.49, 0.70]	0.33	.739		
Group 2	0.16	0.38	[-0.56, 0.87]	0.41	.682		
Group 3	0.10	0.39	[-0.65, 0.85]	0.24	.809		
Time	1.38	0.36	[0.68, 2.07]	3.79	< .001		
Emotion regulation Baseline	0.96	0.03	[0.90, 1.02]	29.36	< .001		
Extraversion	-0.06	0.04	[-0.02, 0.13]	1.40	.164		
Neuroticism	-0.03	0.03	[-0.10, 0.03]	-1.01	.314		
Group 2 X Time	-1.40	0.53	[-2.40, -0.40]	-2.66	.009		
Group 3 X Time	-1.56	0.55	[-2.60, -0.52]	-2.85	.005		
Random Effects							
Participant (Intercept)						0.00	0.00
Residual						1.58	1.26

*Note.* Model equation: ERA ~ Group * Time + ERA_Baseline + E+ N + (1 | VpnCodeScreening). Model fit: R^2^ marginal = 0.87, R^2^ conditional = 0.87.

**Table B5:** Extraversion

Variable	Est/Beta	*SE*	95% CI	*t*	*p*	Variance	*SD*
Fixed Effects							
Intercept	0.87	0.86	[25.65, 31.14]	1.01	.313		
Group 2	0.01	0.60	[-3.75, 4.18]	0.01	.991		
Group 3	-0.03	0.62	[-5.07, 3.18]	-0.05	.963		
Time	1.27	0.58	[0.14, 2.40]	2.18	.031		
Extraversion Baseline	0.97	0.03		36.56	< .001		
Group 2 X Time	-1.16	0.84	[-2.80, 0.48]	-1.37	.172		
Group 3 X Time	-1.88	0.88	[-3.58, -0.17]	-2.14	.034		
Random Effects							
Participant (Intercept)						0.00	0.00
Residual						4.07	2.02

*Note*. Model equation: E ~ Group * Time + E_Baseline + (1 | VpnCodeScreening); Group 2 – affiliation vs. specific goal coaching. Model fit: R^2^ marginal **=** 0.91, R^2^ conditional = 0.91.

**Table B6:** Extraversion (Including Emotion Regulation as Covariate)

Variable	Est/Beta	*SE*	95% CI	*t*	*p*	Variance	*SD*
Fixed Effects							
Intercept	0.52	0.88	[24.05, 30.85]	0.59	.554		
Group 2	0.23	0.61	[-3.70, 5.79]	0.38	.708		
Group 3	0.22	0.64	[-4.40, 5.52]	0.35	.726		
Time	1.27	0.58	[0.14, 2.40]	2.19	.030		
Extraversion Baseline	0.97	0.03		36.75	< .001		
Emotion regulation	0.16	0.10	[-1.17, 0.69]	1.58	.117		
Group 2 X Time	-1.16	0.84	[-2.80, 0.48]	-1.38	.169		
Group 3 X Time	-1.88	0.87	[-3.58, 0.17]	-2.15	.033		
Random Effects							
Participant (Intercept)						0.00	0.00
Residual						4.02	2.01

*Note*. Model equation: E ~ Group * Time + E_Baseline + ERA + (1 | VpnCodeScreening). Model fit: R^2^ marginal = 0.91, R^2^ conditional = 0.91.

**Table B7:** Neuroticism

Variable	Est/Beta	*SE*	95% CI	*t*	*p*	Variance	*SD*
Fixed Effects							
Intercept	0.63	0.73	[17.14, 24.95]	0.86	.390		
Group 2	0.03	0.72	[-6.23, 5.06]	0.04	.970		
Group 3	0.11	0.76	[-2.18, 9.57]	0.14	.890		
Time	-0.60	0.71	[-1.98, 0.77]	-0.85	.390		
Neuroticism Baseline	0.97	0.03		37.45	< .001		
Group 2 X Time	1.45	1.02	[-0.55, 3.44]	1.41	.160		
Group 3 X Time	-0.16	1.06	[-2.23, 1.91]	-0.15	.880		
Random Effects							
Participant (Intercept)						0.00	0.00
Residual						6.01	2.45

*Note*. Model equation: N ~ Group * Time + N_Baseline + (1 | VpnCodeScreening). Model fit: R^2^ marginal = 0.92, R^2^ conditional = 0.92.
